# ACEI-Inhibitory Peptides Naturally Generated in Meat and Meat Products and Their Health Relevance

**DOI:** 10.3390/nu10091259

**Published:** 2018-09-07

**Authors:** Leticia Mora, Marta Gallego, Fidel Toldrá

**Affiliations:** Instituto de Agroquímica y Tecnología de Alimentos (CSIC), Avenue Agustín Escardino 7, 46980 Paterna, Valencia, Spain; lemoso@iata.csic.es (L.M.); mgallego@iata.csic.es (M.G.)

**Keywords:** meat, peptides, antihypertensive, peptidomics

## Abstract

Meat and meat products have been described as a very good source of angiotensin I converting enzyme (ACEI)-inhibitory peptides. The generation of bioactive peptides can occur through the action of endogenous muscular enzymes during processing, gastrointestinal digestion, or by using commercial enzymes in laboratory or industry under controlled conditions. Studies of bioavailability are necessary in order to prove the positive health effect of bioactive peptides in the body as they should resist gastrointestinal digestion, cross the intestinal barrier, and reach blood stream and target organs. However, in order to better understand their effect, interactions, and bioavailability, it is necessary to consider food matrix interactions and continue the development of quantitative methodologies in order to obtain more data that will enable advances in the field of bioactive peptides and the determination of their influence on health.

## 1. Introduction

Bioactive peptides derived from food proteins can exert different effects after their absorption in the human body, such as prevention of diseases or physiological modulation. Physiological properties such as antihypertensive, antioxidant, antithrombotic, or hypocholesterolemic activity in the cardiovascular system [1,2]; mineral binding, antidiabetic, antimicrobial, or anti-inflammatory effects in the gastrointestinal system [3,4]; cytomodulatory or immunomodulatory actions in the immune system [5]; and opioid agonist or antagonist activity in the nervous system [6] have been recently described to be exerted by different food-derived peptides [7].

The activity of angiotensin I-converting enzyme (ACEI) inhibitors has been extensively studied over the last decade. The main reason for this interest is the relevance of hypertension in the development of cardiovascular diseases, which is the most important public health problem of this century. In this respect, different synthetic drugs are available on the market for the treatment of hypertension but their numerous side effects have focused researchers’ interest on the search of alternative non-toxic and naturally generated peptides for controlling blood pressure [8].

**Figure 1:**
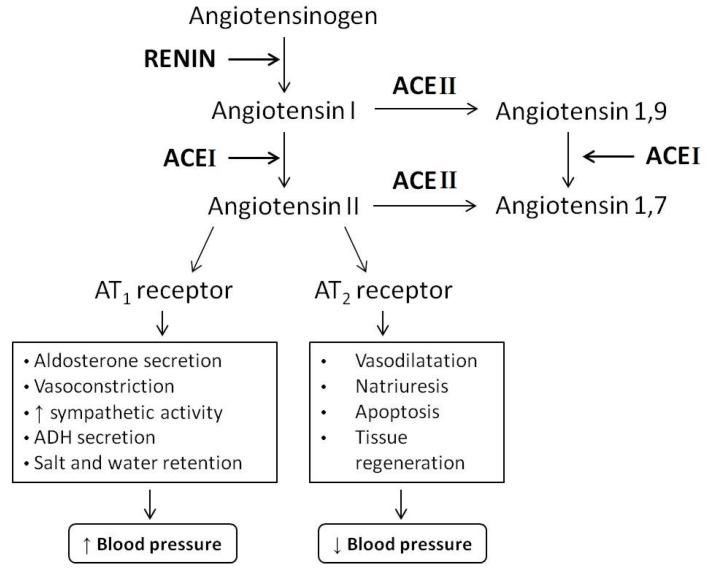
The renin-angiotensin system (RAS). ACEI: angiotensin I-converting enzyme; ACEII: angiotensin II-converting enzyme.

ACEI is a dipeptidyl carboxypeptidase enzyme that participates in the renin–angiotensin system (RAS) and converts angiotensin-I into the vasoconstrictor angiotensin-II by cleaving two amino acids at the same time, thus inactivating the vasodilator bradykinin. The role of ACEI inhibitors is to maintain the balance between the vasoconstrictive and salt-retentive effects of angiotensin-II and vasodilator effects of bradykinin (Figure 1). Thus, the main interest for studying ACEI-inhibitory natural peptides is due to their capacity to inhibit ACEI, which lead to a decrease in blood pressure by inactivating the formation of angiotensin-II [9].

In this article, the generation of ACEI-inhibitory peptides from meat and meat products and their identification by empirical and in silico approaches have been reviewed, as well as the latest studies on bioavailability from the point of view of their health relevance. A discussion about current limitations and challenges to be overcome in order to advance in the state-of-the-art of this field is also included.

## 2. Generation of Meat-Derived ACEI-Inhibitory Peptides

ACEI-inhibitory peptides are usually small peptides with sizes comprising between 2 and 20 amino acids. Their function depends on the protein source, hydrolysis conditions, degree of hydrolysis, molecular mass, and amino acid composition as well as the position of amino acids in the peptide sequences. In this respect, ACEI-inhibitory peptides have been described that have hydrophobic and branched-chain amino acids in their structure. According to the literature, the type of amino acids located in the three positions close to the C-terminal end of the ACEI-inhibitory peptide is important for activity. The presence of aromatic, positively-charged, and basic amino acids in these positions is important for competitive binding to the ACEI active site. In fact, milk-derived tripeptides containing prolines might have different *cis/trans* configurations of bonds which could influence their access/binding to the ACEI complex [10,11,12,13,14]. Milk proteins have been described as a very good source of antihypertensive peptides, released during gastrointestinal digestion or food processing. The tripeptides Ile-Pro-Pro and Val-Pro-Pro, that are released from casein during the fermentation of milk, have been described as antihypertensive in several animal models as well as in clinical studies [15].

**Figure 2:**
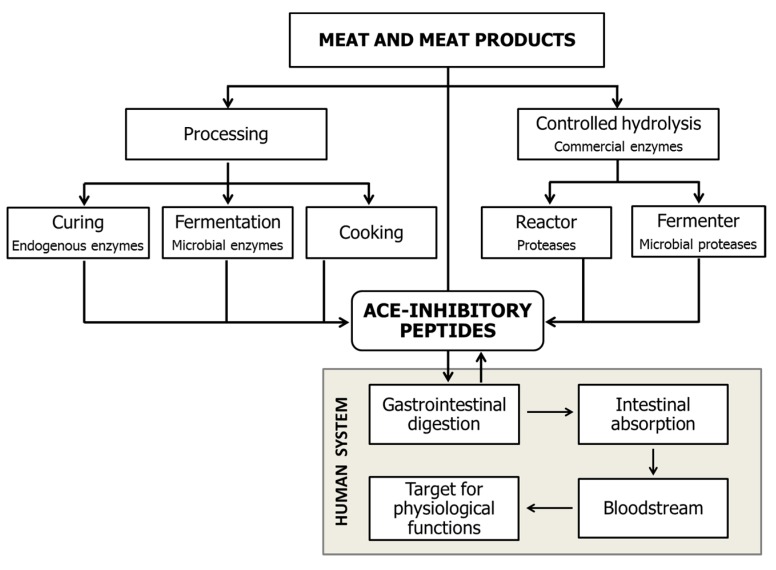
Different ways to generate ACEI-inhibitory peptides.

ACEI-inhibitory peptides obtained from food sources are inactive within the intact parent protein but can exert their activity once they are released by hydrolysis. Different ways of generating ACEI-inhibitory peptides have been utilised, as shown in Figure 2.

### 2.1. Bioactive Peptides Generated during Gastrointestinal Digestion

Gastrointestinal digestion (GI) is the last step for the generation of bioactive peptides from foods. After food ingestion, gastrointestinal peptidases such as pepsin, trypsin, or chymotrypsin are the main proteases responsible for the generation of multiple peptides, including bioactive sequences. In the laboratory, gastrointestinal digestion can be simulated using specific commercial enzymes and controlled conditions of pH and temperature. Thus, a simulated gastrointestinal digestion of raw pork meat using pepsin and pancreatin indicated that the physiological digestion of pork proteins could generate peptides with biological activity [16]. These results were later confirmed in vitro with the ACEI-inhibitory peptides KAPVA (Lys-Ala-Pro-Val-Ala) and PTPVP (Pro-Thr-Pro-Val-Pro), showing half maximal inhibitory concentration (IC_50_) values of 46.56 and 256.41 µM, respectively [17], using the ACEI-inhibitory method described by Sentandreu and Toldrá (2006) [18]. Later, it was confirmed that these peptides also produced in vivo a decrease in the systolic blood pressure (SBP) of spontaneously hypertensive rats (SHRs) of 33.72 ± 8.01 mmHg and 25.66 ± 6.84 mmHg, respectively, after single oral administration of the synthesised peptides dissolved in distilled water at a concentration of 2 mg/mL and adjusted to 1 mg of peptide/kg of body weight administered by gastric intubation. The SBP was measured by the tail cuff method with a programmed electro-sphygmomanometer, and the effect lasted up to 6 h after single administration [19]. A recent study evaluated the digestion of beef proteins by studying the kinetics of peptide release in vivo by regularly sampling the gastric contents using a cannula. The obtained results were evaluated with bioinformatics tools in order to identify potentially bioactive peptides [20].

On the other hand, GI simulation has also been used in studies of bioavailability of certain peptide sequences in order to demonstrate whether they could exert a positive health effect in the body, as they should resist further GI digestion, cross the intestinal barrier, and reach the blood stream and target organs.

Finally, necessary treatments of meat before consumption such as cooking could facilitate the later generation of bioactive peptides due to denatured proteins being more susceptible to be hydrolysed by the enzymes of the intestinal tract.

### 2.2. Hydrolysis Treatments with Commercial Enzymes

The most used methodology for the generation of bioactive peptides is the hydrolysis of proteins with commercial enzymes. Proteases from different sources such as of microbial, plant, or animal origin, have been used for the hydrolysis of food proteins. In meat and meat products, Flavourzyme from *Aspergillus oryzae*, and Neutrase and Alcalase from *Bacillus subtilis* and *Bacillus lincheniformis*, respectively, have been the most used in the generation of bioactive peptides. In addition, proteases from plant origin such as bromelain and papain have been described as interesting enzymes for the hydrolysis of meat proteins by their contribution to meat tenderisation. These enzymes show a wider specificity in comparison with other enzymes such as trypsin or pepsin, cleaving peptide bonds from a wide variety of regions and frequently acting as either endopeptidases, or as exopeptidases hydrolysing amino acids from N- and C-terminal sites. In fact, the activity and hydrolytic specificity of many commercial peptidases is not clearly defined by manufacturers and thus, the degree of hydrolysis and final content of peptides is difficult to predict [8].

Several studies have reported the generation and identification of ACEI-inhibitory peptides resulting from hydrolysates of pork [17,21,22], chicken [23,24], and beef [25]. However, proteins obtained from by-products constitute good substrates that can be used to obtain bioactive peptides through this methodology [26,27], giving an extra added value to these products as well as reducing their environmental impact. In fact, this is the most commonly used procedure when the objective is to obtain high amounts of bioactive peptides for commercialisation, because its efficiency is optimised in a laboratory and later scaled up for pilot plant and industrial production.

### 2.3. Bioactive Peptide Generation during Ageing and the Processing of Meat

Bioactive peptides can be generated through the action of endogenous enzymes in ageing and curing processes as well as in combination with microbial peptidases such as in fermentation processes. Proteolysis by endogenous proteases is the most important phenomena occurring in the ageing of meat that influences its final characteristics with endogenous peptidases as main figures. Broadly speaking, endopeptidases such as calpains and cathepsins are first responsible for the hydrolysis of proteins into large fragments and oligopeptides, which affect the texture of meat during ageing and the initial steps of curing processes. Later, the activity of exopeptidases such as aminopeptidases and carboxypeptidases will generate small peptides and free amino acids, responsible for the characteristic flavour of dry-cured products. Some of the generated small peptides have also been described as bioactive peptides, exerting activities such as ACEI-inhibitory activity and antihypertensive, antioxidant, antilisteria, dipeptidyl peptidase IV (DPP-IV) inhibitory, and anti-inflammatory activity.

Dry-fermented sausages are elaborated using shorter processes with microorganisms such as lactic acid bacteria (LAB) (as a starter), yeasts, or moulds that are responsible for fermentation followed by ripening/drying. Lactic acid bacteria such as *Lactobacillus sakei*, *Lactobacillus curvatus*, *Lactobacillus plantarum* and *Lactobacillus casei* alone or in combination with staphylococci, *Kocuria*, yeast, or moulds, exert proteolysis through the action of endo- and exopeptidases. In general, these fermentation processes are involved in the liberation of small peptides and free amino acids that not only affect flavour development but also contribute to the generation of bioactive peptides [28].

The presence of ACEI-inhibitory peptides naturally generated during the processing of meat products such as dry-cured hams or dry-fermented sausages has also been described [29,30,31,32,33].

## 3. Identification of ACEI-Inhibitory Peptides

Traditionally, empirical approaches have been the method of choice for the identification of bioactive peptides from food matrices. However, it is very challenging when the objective is to generate specific peptide sequences that are able to exert certain activity. Then, the experimental design can be simplified by using bioinformatics for computer simulation in silico.

**Figure 3:**
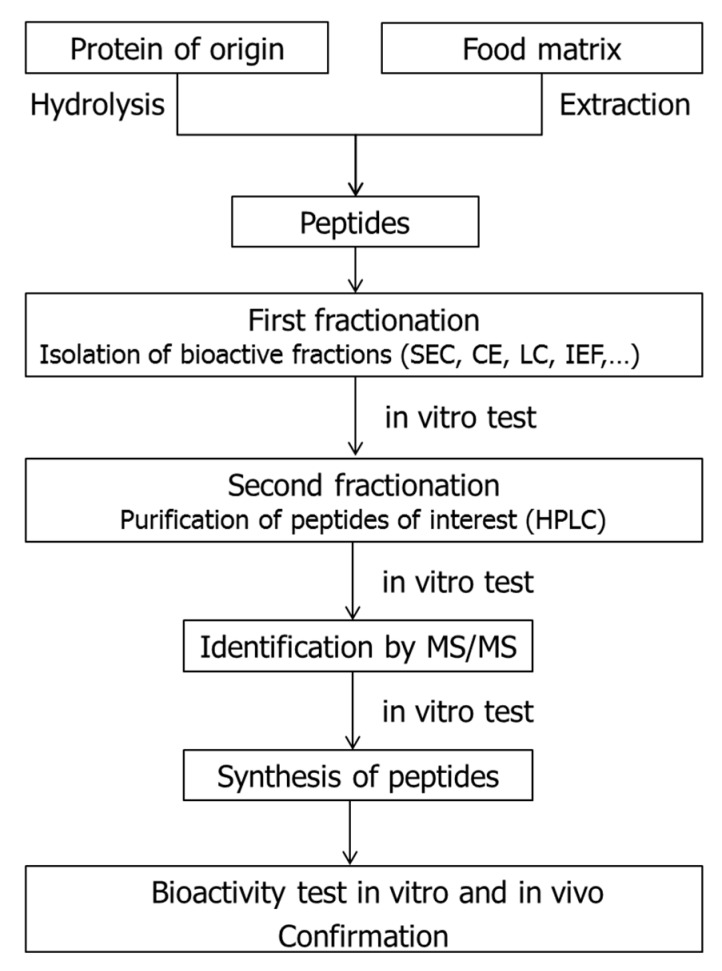
Scheme of the traditional empirical procedure for the identification and confirmation of bioactive peptides from food matrices. SEC: size-exclusion chromatography; CE: capillary electrophoresis; LC: liquid chromatography; IEF: isolectric focusing; HPLC: high performance liquid chromatography; MS/MS: mass spectrometry in tandem.

Empirical approaches used for the identification of bioactive peptides including ACEI-inhibitory peptides in complex sample matrices such as meat and meat products involve: (1) the release of the bioactive sequences from the parent protein; (2) preliminary in vitro assays to screen for bioactivity; (3) purification and separation through the use of high-resolution techniques, such as chromatography; (4) additional in vitro assays to determine the most active fractions; (5) identification of peptides by mass spectrometry (MS) techniques; (6) selection and synthesis of potential bioactive peptides; and (7) in vitro and in vivo confirmation of the bioactivity [34]. A scheme of the traditional empirical procedure for the identification and confirmation of bioactive peptides from food matrices is shown in Figure 3.

In vitro ACEI-inhibitory activity is typically measured by monitoring the conversion of a specific substrate by ACEI in the presence and absence of inhibitors. Spectrophotometric and chromatographic methods have been commonly used to measure the hydrolysis of substrates such as Hippuryl-His-Leu (HHL) or the fluorogenic *o*-aminobenzoylglycyl-*p*-nitrophenylalanylproline. However, the inhibitory activities of these peptides on ACEI activity do not always correlate with antihypertensive effects. In this regard, SHRs are the animal model most frequently used to verify the in vivo efficacy of ACEI-inhibitory peptides. Some studies have evaluated the effects on SBP of SHRs after oral administration of meat hydrolysates or peptide extracts showing ACEI-inhibitory activity [19,35,36,37]. Table 1 shows the antihypertensive effects of meat-derived peptides after single oral administration to SHR [38,39,40,41,42,43,44]. As a last step, human clinical trials are the most accurate method to assess the efficacy and physiological functions of meat-derived antihypertensive peptides, although few studies have been done in this respect due to the complexity and expensive costs. Hodgson et al. (2006) suggested that a partial substitution of carbohydrate intake with protein-rich foods such as lean red meat may lower SBP in hypertensive persons [45], whereas a clinical study done by Saiga-Egusa et al. (2009) using chicken collagen hydrolysate observed a SBP reduction in mildly hypertensive subjects by inhibiting ACEI and plasma renin activity [46]. Additionally, it has been reported that the regular consumption of dry-cured ham would not increase blood pressure despite its high salt content, and even could exert other beneficial effects on cardiovascular health related to glucose and lipid metabolism, and inflammatory processes [47,48,49].

**Figure 4:**
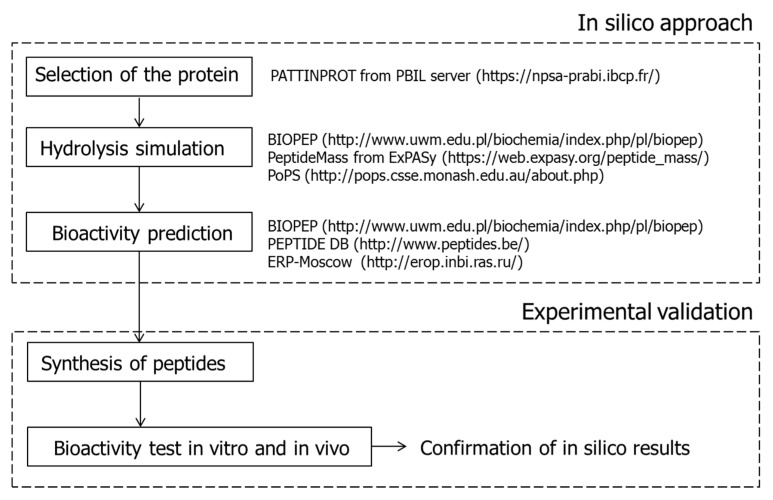
Main steps of in silico approaches and open access databases for the selection of the protein, hydrolysis simulation and bioactivity prediction.

In addition to empirical approaches, the use of in silico analyses that combine bioinformatics tools and peptide databases has been increasingly used as a cost- and time-effective alternative. This predictive strategy enables to obtain biological and chemometric information on peptide sequences to be obtained through a series of steps: (1) selection of proteins of interest with known amino acid sequences by predicting their potential as precursors of novel bioactive peptides; (2) in silico protein digestion by selected proteolytic enzymes; (3) in silico identification and characterisation of peptides; (4) bioactivity prediction using a combination of sequence biochemical properties and databases of known bioactive peptides; (5) peptide synthesis; and (6) in vitro or in vivo confirmation of the bioactivity [50]. Figure 4 shows the main steps of in silico approaches and suggests open access databases and bioinformatics tools for the selection of the protein, hydrolysis simulation and bioactivity prediction. In this regard, BIOPEP is a widely used database for the study and identification of food-derived bioactive peptides as well as for in silico digestion and prediction of their bioactivities. In addition, computational models such as quantitative structure-activity relationships (QSAR), quantitative structure-property relationships (QSPR), and molecular docking simulations allow the discovery and characterisation of structural and physical-chemical properties such as hydrophilicity-hydrophobicity, molecular size, and electronic and steric characteristics, and results in very useful information to evaluate the potential affinity between the biopeptide sequence of interest and the target [50,51].

In silico approaches have shown that some bovine, porcine and chicken proteins such as collagen, connectin, and myosin are good sources of ACEI-inhibitory peptides, which could be released from the parent protein through the action of determined enzymes [52,53,54]. Moreover, computer simulations are fundamental for understanding molecular mechanisms and ACEI-peptide interactions such as the fact that the C-terminal tripeptide sequence, hydrophobicity, and positive charge of the amino acid residues in this region of the peptide have a major influence on ACEI inhibition [55].

At the end, empirical and in silico approaches converge in the need for the confirmation of both the identity of the generated/predicted peptide sequences and their activity in the complexity of the matrix (see Figure 4).

## 4. Bioavailability of ACEI-Inhibitory Peptides

Bioavailability studies are necessary to assess whether the bioactive peptide can reach its target site in active form and sufficient quantity to exert health effects in the organism. The action of gastrointestinal enzymes, intestinal absorption, cellular uptake, and action of blood plasma peptidases can modify the structure of ACEI-inhibitory peptides or hydrolyse them leading to a loss, maintenance, or gain of bioactivity [11,56].

The use of digestion models and LC-MS techniques combined with in silico and in vitro/in vivo approaches have enabled evaluation of the stability of ACEI-inhibitory peptides from beef, chicken and pork meat in gastrointestinal digestion as well as the identification and quantification of the resulting products [17,20,30,57,58,59]. On the other hand, cell models such as heterogeneous human epithelial colorectal adenocarcinoma cells (Caco-2 cell) monolayers have been useful to study the transepithelial transport of ACEI-inhibitory peptides derived from meat proteins, being able to determine structural changes and amount of peptides transported or the involved transport pathway [60,61,62]. The ability of peptides to resist enzymatic degradation and be transported across intestinal membranes into blood circulation depends on their characteristics, length, and amino acid composition. In this regard, proline-rich peptides are more resistant to be attacked by gastrointestinal enzymes, and di- and tri-peptides could be absorbed intact by peptide transporter systems and hydrolysed later [63]. The low transport ability of oligopeptides compared to di- and tri-peptides is probably due to their length and involve paracellular route, while the hydrophobicity of peptides does not seem to influence absorption [64]. Additionally, the absorption of peptides could be affected by co-existing peptides and food components, which can share the transport pathway or participate in its regulation [64].

The bioavailability and bioaccessibility of bioactive peptides can also be affected by processing/storage conditions and food matrix-peptide interactions that can lead to peptide modifications with changes in its native structure and activity [65]. Several studies have evaluated the stability of ACEI-inhibitory peptides after household cooking preparations of pork and beef meat [66], different temperatures and pH used when processing meat products [30,67], and the effect of ageing under industrial conditions (vacuum-packed and chilled-storage) and cooking of beef meat [68].

## 5. Challenges and Limitations

Currently, with the basis of knowledge for the identification of bioactive peptides already clear, it is necessary to continue the research in bioactive peptides to achieve a better understanding of their effect, interactions, and bioavailability. In this sense, several authors have established the need for serious consideration of food matrix interactions, especially when the objective is to use the bioactive peptides as a functional ingredient [65]. Increasingly, once the peptide has been identified in a food matrix, it is synthesised and characterised as an individual molecule. However, the expected in vitro and/or in vivo activity may differ when the peptide interacts with the complex mixture of compounds that are taking part of any food.

On the other hand, increased effort on the development of quantitative methodologies for a better understanding of hydrolysis, bioactivity, and/or bioavailability is necessary. Data such as the quantity of specific naturally generated peptides in the original food and the dose of a bioactive peptide needed to exert an effect in vivo, as well as the final sequences and amount present in bloodstream and target organ after GI digestion are key data for advancing in the field of bioactive peptides and their health influence. In fact, determining the quantity of ACEI-inhibitory peptides in the meat sample that are able to reach the target site in the human system is of fundamental importance in bioavailability studies to better understand the effects and mechanisms of action of these peptides. The main limitation for quantitation is the nature of sample: small peptides often comprise fewer than four amino acids at low abundance, and there is high complexity of the matrix [69]. Current advances in mass spectrometry instrumentation, bioinformatics tools, and updated protein databases are contributing to progress in quantitative peptidomics [68].

## 6. Conclusions

Meat and meat products have been described as a very good source of ACEI-inhibitory peptides. With proteins being a major constituent of meats, the generation of bioactive peptides from meat proteins has been described as occurring either through the action of endogenous muscular enzymes during processing, during GI digestion, or by using commercial enzymes in the laboratory or in industrial processes under controlled conditions. The identification of ACEI-inhibitory peptides has been traditionally done using empirical approaches, although currently there is an increasing interest in in silico approaches based on bioinformatics as they are less time-consuming and cheaper methodologies. However, despite the identification of bioactive peptides being clear, there is an increasing need to study food matrix interactions, especially when the objective is to use the bioactive peptides as a functional ingredient. The quantitation of these peptides for a better understanding of their health influence and bioavailability is necessary to advance in this field.

## Figures and Tables

**Table 1:** Angiotensin I-converting enzyme (ACEI)-inhibitory peptides identified in meat and meat products with antihypertensive effects in spontaneously hypertensive rats.

Source	Peptide Sequence	Parent Protein	Hydrolysis Treatment	IC_50_ (µM) ^a^	Dose (mg/kg BW) ^b^	SBP (mmHg) ^c^	Time (h) ^d^	Reference
Chicken muscle	IKW	—	Thermolysin	0.21	60	−0.17	4	[23]
Chicken muscle	LKP	Aldolase	Thermolysin	0.32	60	−0.18	4	[23]
Chicken muscle	FKGRYYP	Creatine kinase	Thermolysin	0.55	60	0	—	[23]
Chicken muscle	GA(Hyp)GL(Hyp)GP	Collagen	Proteases	29.4	4.5	−0.18	6	[39]
Chicken bone	YYRA	Inmunoglobin heavy chain	Pepsin	57.2	10	−0.20	6	[40]
Porcine muscle	MNPPK	Myosin	Thermolysin	945.5	1	−0.23	6	[35]
Porcine muscle	ITTNP	Myosin	Thermolysin	549	1	−0.21	6	[35]
Porcine muscle	VKKVLGNP	Myosin light chain	Pepsin	29	10	−0.24	3	[41]
Porcine muscle	KRQKYDI	Troponin	Pepsin	26.2	10	−0.9	6	[42]
Porcine muscle	KRVITY	Myosin heavy chain	Pepsin	6.1	10	−0.23	6	[43]
Porcine muscle	VKAGF	Actin	Pepsin	20.3	10	−0.17	6	[43]
Porcine muscle	RPR	Nebulin	Pepsin + pancreatin	382	1	−0.33	6	[19]
Porcine muscle	KAPVA	Titin	Pepsin + pancreatin	46.56	1	−0.33	6	[19]
Porcine muscle	PTPVP	Titin	Pepsin + pancreatin	256.41	1	−0.25	6	[19]
Porcine skin	GF(Hyp)GP	Collagen	*Aspergillus* protease	91	10	−0.20	8	[44]
Goat muscle	FQPS	—	Protamex^®^ + Flavourzyme^®^	27.0	2.39	−0.10	8	[45]
Spanish dry-cured ham	AAATP	Allantoicase	No treatment	100	1	−0.26	8	[19]

^a^ IC_50_ value is the peptide concentration that inhibits 50% of ACE activity; ^b^ Oral administration of the peptide expressed as mg/kg body weight of rat; ^c^ Maximum decrease in systolic blood pressure (SBP) after administration of the peptide to spontaneously hypertensive rats; ^d^ Time after peptide administration to exert the maximum decrease in systolic blood pressure.

## References

[1] Gallego M., Mora L., Toldrá F. (2018). Health relevance of antihypertensive peptides in foods. Curr. Opin. Food Sci..

[2] Lorenzo J.M., Munekata P.E.S., Gómez B., Barba F.J., Mora L., Pérez-Santaescolástica C., Toldrá F. (2018). Bioactive peptides as natural antioxidants in food products—A review. Trends Food Sci. Technol..

[3] Santos J.C.P., Sousa R.C.S., Otoni C.G., Moraes A.R.F., Souza V.G.L., Medeiros E.A.A., Espitia P.J.P., Pires A.C.S., Coimbra J.S.R., Soares N.F.F. (2018). Nisin and other antimicrobial peptides: Production, mechanisms of action, and application in active food packaging. Innov. Food Sci. Emerg. Technol..

[4] Moughan P.J., Rutherfurd-Markwick K., Calder P.C., Parveen Y. (2013). Food bioactive proteins and peptides: antimicrobial, immunomodulatory and anti-inflammatory effects. Diet, Immunity and Inflammation.

[5] Chalamaiah M., Yu W., Wu J. (2018). Immunomodulatory and anticancer protein hydrolysates (peptides) from food proteins: A review. Food Chem..

[6] Arısoy S., Üstün-Aytekin Ö. (2018). Hydrolysis of food-derived opioids by dipeptidyl peptidase IV from *Lactococcus lactis* spp. *Lactis*. Food Res. Int..

[7] Mora L., Aristoy M.C., Toldrá F., Varelis P., Melton L., Shahidi F. (2018). Chapter Bioactive peptides. Encyclopedia of Food Chemistry.

[8] Toldrá F., Reig M., Aristoy M.C., Mora L. (2018). Generation of bioactive peptides during processing. Food Chem..

[9] Wu J., Liao W., Udenigwe C.C. (2017). Revisiting the mechanisms of ACE inhibitory peptides from food proteins. Trends Food Sci. Technol..

[10] Girgih A.T., He R., Malomo S., Offengenden M., Wu J., Aluko R.E. (2014). Structural and functional characterization of hemp seed (*Cannabis sativa* L.) protein derived antioxidant and antihypertensive peptides. J. Funct. Foods.

[11] Hernández-Ledesma B., del Mar Contreras M., Recio I. (2011). Antihypertensive peptides: Production, bioavailability and incorporation into foods. Adv. Colloid Interface Sci..

[12] Lassoued I., Mora L., Nasri R., Jridi M., Toldrá F., Aristoy M.-C., Nasri M. (2015). Characterization and comparative assessment of antioxidant and ACE inhibitory activities of thornback ray gelatin hydrolysates. J. Funct. Foods.

[13] Shahidi F., Zhong Y. (2008). Bioactive peptides. J. AOAC Int..

[14] Zheng Y., Li Y., Zhang Y., Ruan X., Zhang R. (2017). Purification, characterization, synthesis, in vitro ACE inhibition and in vivo antihypertensive activity of bioactive peptides derived from oil palm kernel glutelin-2 hydrolysates. J. Funct. Foods.

[15] Jäkälä P., Vapaatalo H. (2010). Antihypertensive peptides from milk proteins. Pharmaceuticals.

[16] Escudero E., Sentandreu M.A., Toldrá F. (2010). Characterization of peptides released by in vitro digestion of pork meat. J. Agric. Food Chem..

[17] Escudero E., Sentandreu M.A., Arihara K., Toldrá F. (2010). Angiotensin I converting enzyme inhibitory peptides generated from in vitro gastrointestinal digestion of pork meat. J. Agric. Food Chem..

[18] Sentandreu M.A., Toldrá F. (2006). A rapid, simple and sensitive fluorescence method for the assay of angiotensin-I converting enzyme. Food Chem..

[19] Escudero E., Toldrá F., Sentandreu M.A., Nishimura H., Arihara K. (2012). Antihypertensive activity of peptides identified in the in vitro gastrointestinal digest of pork meat. Meat Sci..

[20] Sayd T., Dufour C., Chambon C., Buffière C., Remond D., Santé-Lhoutellier V. (2018). Combined in vivo and in silico approaches for predicting the release of bioactive peptides from meat digestion. Food Chem..

[21] Arihara K., Nakashima Y., Mukai T., Ishikawa S., Itoh M. (2001). Peptide inhibitors for angiotensin I-converting enzyme from enzymatic hydrolysates of porcine skeletal muscle proteins. Meat Sci..

[22] Katayama K., Tomatsu M., Fuchu H., Sugiyama M., Kawahara S., Yamauchi K., Kawamura Y., Muguruma M. (2003). Purification and characterization of an angiotensin I-converting enzyme inhibitory peptide derived from porcine troponin C. Anim. Sci. J..

[23] Fujita H., Yokoyama K., Yoshikawa M. (2000). Classification and antihypertensive activity of angiotensin I-converting enzyme inhibitory peptides derived from food proteins. J. Food Sci..

[24] Terashima M., Baba T., Ikemoto N., Katayama M., Morimoto T., Matsumura S. (2010). Novel angiotensin-converting enzyme (ACE) inhibitory peptides derived from boneless chicken leg meat. J. Agric. Food Chem..

[25] Jang A., Lee M. (2005). Purification and identification of angiotensin converting enzyme inhibitory peptides from beef hydrolysates. Meat Sci..

[26] Adje E.Y., Balti R., Guillochon D., Nedjar-Arroume N. (2011). α 67–106 of bovine hemoglobin: A new family of antimicrobial and angiotensin I-converting enzyme inhibitory peptides. Eur. Food Res. Technol..

[27] Banerjee P., Shanthi C. (2012). Isolation of novel bioactive regions from bovine Achilles tendon collagen having angiotensin I-converting enzyme-inhibitory properties. Process Biochem..

[28] Mora L., Gallego M., Escudero E., Reig M., Aristoy M.C., Toldrá F. (2015). Small peptides hydrolysis in dry-cured meats. Int. J. Food Microbiol..

[29] Escudero E., Mora L., Fraser P.D., Aristoy M.C., Arihara K., Toldrá F. (2013). Purification and identification of antihypertensive peptides in Spanish dry-cured ham. J. Proteomics.

[30] Escudero E., Mora L., Toldrá F. (2014). Stability of ACE inhibitory ham peptides against heat treatment and in vitro digestion. Food Chem..

[31] Mora L., Escudero E., Toldrá F. (2016). Characterization of the peptide profile in Spanish Teruel, Italian Parma and Belgian dry-cured hams and its potential bioactivity. Food Res. Int..

[32] Mejri L., Vásquez-Villanueva R., Hassouna M., Marina M.L., García M.C. (2017). Identification of peptides with antioxidant and antihypertensive capacities by RP-HPLC-Q-TOF-MS in dry fermented camel sausages inoculated with different starter cultures and ripening times. Food Res. Int..

[33] Gallego M., Mora L., Escudero E., Toldrá F. (2018). Bioactive peptides and free amino acids profiles in different types of European dry-fermented sausages. Int. J. Food Microbiol..

[34] Sánchez-Rivera L., Martínez-Maqueda D., Cruz-Huerta E., Miralles B., Recio I. (2014). Peptidomics for discovery, bioavailability and monitoring of dairy bioactive peptides. Food Res. Int..

[35] Nakashima Y., Arihara K., Sasaki A., Mio H., Ishikawa S., Itoh M. (2002). Antihypertensive activities of peptides derived from porcine skeletal muscle myosin in spontaneously hypertensive rats. J. Food Sci..

[36] Saiga A.I., Iwai K., Hayakawa T., Takahata Y., Kitamura S., Nishimura T., Morimatsu F. (2008). Angiotensin I-converting enzyme-inhibitory peptides obtained from chicken collagen hydrolysate. J. Agric. Food Chem..

[37] Mora L., Escudero E., Arihara K., Toldrá F. (2015). Antihypertensive effect of peptides naturally generated during Iberian dry-cured ham processing. Food Res. Int..

[38] Iwai K., Saiga-Egusa A., Hayakawa T., Shimizu M., Takahata Y., Morimatsu F. (2008). An angiotensin I-converting enzyme (ACE)-inhibitory peptide derived from chicken collagen hydrolysate lowers blood pressure in spontaneously hypertensive rats. J. Jpn. Soc. Food Sci..

[39] Nakade K., Kamishima R., Inoue Y., Ahhmed A., Kawahara S., Nakayama T., Maruyama M., Numata M., Ohta K., Aoki T. (2008). Identification of an antihypertensive peptide derived from chicken bone extract. Anim. Sci. J..

[40] Katayama K., Mori T., Kawahara S., Miake K., Kodama Y., Sugiyama M., Kawamura Y., Nakayama T., Maruyama M., Muguruma M. (2007). Angiotensin-I converting enzyme inhibitory peptide derived from porcine skeletal muscle myosin and its antihypertensive activity in spontaneously hypertensive rats. J. Food Sci..

[41] Katayama K., Anggraeni H.E., Mori T., Ahhmed A.M., Kawahara S., Sugiyama M., Nakayama T., Maruyama M., Muguruma M. (2008). Porcine skeletal muscle troponin is a good source of peptides with angiotensin-I converting enzyme inhibitory activity and antihypertensive effects in spontaneously hypertensive rats. J. Agric. Food Chem..

[42] Muguruma M., Ahhmed A.M., Katayama K., Kawahara S., Maruyama M., Nakamura T. (2009). Identification of pro-drug type ACE inhibitory peptide sourced from porcine myosin B: Evaluation of its antihypertensive effects in vivo. Food Chem..

[43] Ichimura T., Yamanaka A., Otsuka T., Yamashita E., Maruyama S. (2009). Antihypertensive effect of enzymatic hydrolysate of collagen and Gly-Pro in spontaneously hypertensive rats. Biosci. Biotechnol. Biochem..

[44] Mirdhayati I., Hermanianto J., Wijaya C.H., Sajuthi D., Arihara K. (2016). Angiotensin converting enzyme (ACE) inhibitory and antihypertensive activities of protein hydrolysate from meat of Kacang goat (*Capra aegagrus hircus*). J. Sci. Food Agric..

[45] Hodgson J.M., Burke V., Beilin L.J., Puddey I.B. (2006). Partial substitution of carbohydrate intake with protein intake from lean red meat lowers blood pressure in hypertensive persons. Am. J. Clin. Nutr..

[46] Saiga-Egusa A., Iwai K., Hayakawa T., Takahata Y., Morimatsu F. (2009). Antihypertensive effects and endothelial progenitor cell activation by intake of chicken collagen hydrolysate in pre- and mild-hypertension. Biosci. Biotechnol. Biochem..

[47] López M.R.C., Bes-Rastrollo M., Zazpe I., Martínez J.A., Cuervo M., Martínez-González M.A. (2009). Consumo de jamón curado e incidencia de episodios cardiovasculares, hipertensión arterial o ganancia de peso. Med. Clin..

[48] Montoro-García S., Zafrilla-Rentero M.P., Celdrán-de Haro F.M., Piñero-de Armas J.J., Toldrá F., Tejada-Portero L., Abellán-Alemán J. (2017). Effects of dry-cured ham rich in bioactive peptides on cardiovascular health: A randomized controlled trial. J. Funct. Foods.

[49] Martínez-Sánchez S.M., Minguela A., Prieto-Merino D., Zafrilla-Rentero M.P., Abellán-Alemán J., Montoro-García S. (2017). The effect of regular intake of dry-cured ham rich in bioactive peptides on inflammation, platelet and monocyte activation markers in humans. Nutrients.

[50] Agyei D., Ongkudon C.M., Wei C.Y., Chan A.S., Danquah M.K. (2016). Bioprocess challenges to the isolation and purification of bioactive peptides. Food Bioprod. Process..

[51] Carrasco-Castilla J., Hernández-Álvarez A.J., Jiménez-Martínez C., Gutiérrez-López G.F., Dávila-Ortiz G. (2012). Use of proteomics and peptidomics methods in food bioactive peptide science and engineering. Food Eng. Rev..

[52] Gu Y., Majumder K., Wu J. (2011). QSAR-aided in silico approach in evaluation of food proteins as precursors of ACE inhibitory peptides. Food Res. Int..

[53] Minkiewicz P., Dziuba J., Michalska J. (2011). Bovine meat proteins as potential precursors of biologically active peptides-a computational study based on the BIOPEP database. Food Sci. Technol. Int..

[54] Lafarga T., O’Connor P., Hayes M. (2014). Identification of novel dipeptidyl peptidase-IV and angiotensin-I-converting enzyme inhibitory peptides from meat proteins using in silico analysis. Peptides.

[55] Pripp A.H., Isaksson T., Stepaniak L., Sørhaug T. (2004). Quantitative structure-activity relationship modelling of ACE-inhibitory peptides derived from milk proteins. Eur. Food Res. Technol..

[56] Vermeirssen V., Van Camp J., Verstraete W. (2004). Bioavailability of angiotensin I converting enzyme inhibitory peptides. Br. J. Nutr..

[57] Mora L., Bolumar T., Heres A., Toldrá F. (2017). Effect of cooking and simulated gastrointestinal digestion on the activity of generated bioactive peptides in aged beef meat. Food Funct..

[58] Sangsawad P., Roytrakul S., Yongsawatdigul J. (2017). Angiotensin converting enzyme (ACE) inhibitory peptides derived from the simulated in vitro gastrointestinal digestion of cooked chicken breast. J. Funct. Foods.

[59] Dellafiora L., Paolella S., Dall’Asta C., Dossena A., Cozzini P., Galaverna G. (2015). Hybrid in silico/in vitro approach for the identification of angiotensin I converting enzyme inhibitory peptides from Parma dry-cured ham. J. Agric. Food Chem..

[60] Fu Y., Young J.F., Rasmussen M.K., Dalsgaard T.K., Lametsch R., Aluko R.E., Therkildsen M. (2016). Angiotensin I–converting enzyme–inhibitory peptides from bovine collagen: Insights into inhibitory mechanism and transepithelial transport. Food Res. Int..

[61] Gallego M., Grootaert C., Mora L., Aristoy M.C., Van Camp J., Toldrá F. (2016). Transepithelial transport of dry-cured ham peptides with ACE inhibitory activity through a Caco-2 cell monolayer. J. Funct. Foods.

[62] Sangsawad P., Choowongkomon K., Kitts D.D., Chen X.M., Li-Chan E.C., Yongsawatdigul J. (2018). Transepithelial transport and structural changes of chicken angiotensin I-converting enzyme (ACE) inhibitory peptides through Caco-2 cell monolayers. J. Funct. Foods.

[63] Segura-Campos M., Chel-Guerrero L., Betancur-Ancona D., Hernandez-Escalante V.M. (2011). Bioavailability of bioactive peptides. Food Rev. Int..

[64] Shen W., Matsui T. (2017). Current knowledge of intestinal absorption of bioactive peptides. Food Funct..

[65] Udenigwe C.C., Fogliano V. (2017). Food matrix interaction and bioavailability of bioactive peptides: Two faces of the same coin?. J. Funct. Foods.

[66] Jensen I.J., Dort J., Eilertsen K.E. (2014). Proximate composition, antihypertensive and antioxidative properties of the semimembranosus muscle from pork and beef after cooking and in vitro digestion. Meat Sci..

[67] Fu Y., Young J.F., Dalsgaard T.K., Therkildsen M. (2015). Separation of angiotensin I-converting enzyme inhibitory peptides from bovine connective tissue and their stability towards temperature, pH and digestive enzymes. Int. J. Food Sci. Technol..

[68] Mora L., Gallego M., Reig M., Toldrá F. (2017). Challenges in the quantitation of naturally generated bioactive peptides in processed meats. Trends Food Sci. Technol..

[69] Arroume N., Froidevaux R., Kapel R., Cudennec B., Ravallec R., Flahaut C., Bazinet L., Jacques P., Dhulster P. (2016). Food peptides: Purification, identification and role in the metabolism. Curr. Opin. Food Sci..

